# Electroacupuncture Reduces Carrageenan- and CFA-Induced Inflammatory Pain Accompanied by Changing the Expression of Nav1.7 and Nav1.8, rather than Nav1.9, in Mice Dorsal Root Ganglia

**DOI:** 10.1155/2013/312184

**Published:** 2013-03-19

**Authors:** Chun-Ping Huang, Hsiang-Ni Chen, Hong-Lin Su, Ching-Liang Hsieh, Wei-Hsin Chen, Zhen-Rung Lai, Yi-Wen Lin

**Affiliations:** ^1^Department of Life Sciences, National Chung Hsing University, Taichung, Taiwan; ^2^Graduate Institute of Acupuncture Science, China Medical University, 91 Hsueh-Shih Road, Taichung 40402, Taiwan; ^3^Division of Chinese Medicine, China Medical University Beigang Hospital, Yunlin, Taiwan; ^4^Acupuncture Research Center, China Medical University, Taichung, Taiwan; ^5^Graduate Institute of Biotechnology, National Chung Hsing University, Taichung, Taiwan; ^6^Department of Nursing, College of Medicine & Nursing, HungKuang University, Taichung, Taiwan

## Abstract

Several voltage-gated sodium channels (Navs) from nociceptive nerve fibers have been identified as important effectors in pain signaling. The objective of this study is to investigate the electroacupuncture (EA) analgesia mechanism by changing the expression of Navs in mice dorsal root ganglia (DRG). We injected carrageenan and complete Freund's adjuvant (CFA) into the mice plantar surface of the hind paw to induce inflammation and examined the antinociception effect of EA at the Zusanli (ST36) acupoint at 2 Hz low frequency. Mechanical hyperalgesia was evaluated by using electronic von Frey filaments, and thermal hyperalgesia was assessed using Hargreaves' test. Furthermore, we observed the expression and quality of Navs in DRG neurons. Our results showed that EA reduced mechanical and thermal pain in inflammatory animal model. The expression of Nav1.7 and Nav1.8 was increased after 4 days of carrageenan- and CFA-elicited inflammatory pain and further attenuated by 2 Hz EA stimulation. The attenuation cannot be observed in Nav1.9 sodium channels. We demonstrated that EA at Zusanli (ST36) acupoint at 2 Hz low-frequency stimulation attenuated inflammatory pain accompanied by decreasing the expression of Nav1.7 and 1.8, rather than Nav1.9, sodium channels in peripheral DRG neurons.

## 1. Introduction

Recently, several studies have implied that voltage-gated sodium channels (Navs) might be involved in the development of hyperalgesia produced by inflammation [1–3]. Sensory neurons innervating the muscles are considered to sense muscle pain, and Navs are reported to participate in the process of inflammatory pain. Intraplantar injection of carrageenan and CFA is well documented to produce edema, as well as mechanical and thermal hyperalgesia, and has often been used as an inflammatory pain model [1–4]. Sodium channel-induced currents have been identified in CNS neurons [3] and in DRG (dorsal root ganglia) neurons [2] which significantly influence the threshold for action potential firing.

Several voltage-gated sodium channels, Nav1.1, Nav1.3, Nav1.6, Nav1.7, Nav1.8, and Nav1.9, have been reported to express in DRG. Of these Navs, Nav1.8 and Nav1.9 have been reported to demonstrate resistance to TTX (tetrodotoxin), regarded as TTX-R (TTX-resistant) [2–5]. Nav1.7, Nav1.8, and Nav1.9 are usually reported to be participating in inflammation pain and regulating neuron excitability [4]. Notably, previous animal studies have indicated that Nav1.3, Nav1.7, and Nav1.8 play prominent roles in inflammatory pain and can be potentiated by microinjection of carrageenan and CFA into intraplantar [1]. Studies on humans have shown that Nav1.7 is crucial for physiological pain sensations, and mutational alterations to Nav1.7 can result in severe chronic pain sensations [6].

Acupuncture has been widely used for over 3000 years and has been based on the principles of traditional Chinese medicine. Acupuncture is known to stimulate the A*δ*-fibers [7] and modulate pain sensation by activating C-fibers through the meridian [8]. Acupuncture can be used therapeutically to treat diseases systematically [9]. The analgesic effect of acupuncture is already widely accepted. Several studies have suggested that acupuncture increases the release of endogenous opiates [10], serotonin [11], and adenosine to reduce pain [12]. Low-frequency electroacupuncture at 2 Hz induces enkephalins release to activate *μ*-receptor. In contrast, high-frequency stimulation releases dynorphins to activate *κ*-receptors [10].

Navs blockers are usually used for anesthesia and analgesia. To seek for more specific inhibitor with low side effects is possible. The rationale of this study is that Nav1.7, Nav1.8, and Nav1.9 are well known in inflammatory pain. The current study examines the crucial role of Navs and the effect of 2 Hz EA in mechanical and thermal hyperalgesia induced by carrageenan and CFA. We hypothesized that EA could alter expressions of Navs in both carrageenan- and CFA-induced inflammatory pain.

## 2. Methods

### 2.1. Animals and EA Pretreatment

Adult ICR female mice aged 8 to 12 weeks were used in the experiment. The usage of these animals was approved by the *Institute of Animal Care and Use Committee of China Medical University* (Permit no. 101-116-N),* Taiwan*, following the *Guide for the use of Laboratory Animals* (National Academy Press). EA treatment was applied using stainless steel needles (12 mm, 32 G, Yu Kuang, Taiwan) which were inserted into the muscle layer to a depth of 2-3 mm at ST36 acupoint, which is therapeutic in both animal models and clinical study [11]. EA was administered immediately after the injection of carrageenan or CFA and performed every day at the same time (12:00–14:00). A Trio-300 (Japan) stimulator delivered electrical square pulses for 20 min with a 100 *μ*s duration and a 2 Hz frequency. The stimulation amplitude was 1 mA. The same treatment was given to nonacupoint (the upper lateral gluteal muscle but not GB30 acupoint) to be set as the sham control group entitled S-GM [13]. Another sham control group, entitled S-Acu, was induced by needling into ST36 acupoint without manipulation [14, 15].

### 2.2. Inflammatory Pain Models

Mice were anesthetized with 1-2% isoflurane and administered a single injection of 20 *μ*L saline (pH 7.4, buffered with 20 mM HEPES), CFA (0.5 mg/mL heat-killed *M. tuberculosis* Sigma, St. Louis, MO, MSA), or 3% carrageenan (lambda carrageenan and CFA, type IV; Sigma) in the plantar surface of the hind paw to induce intraplantar inflammation. Behavior tests were conducted at day 4 after induction of inflammation, and DRGs were harvested after behavior tests.

### 2.3. Animal Behavior of Mechanical and Thermal Hyperalgesia

Mechanical sensitivities were tested at 4 days after intraplantar injections. All experiments were performed at 30 min after EA (room temperature was approximately 25°C). Mechanical sensitivity was measured by testing the force of responses to stimulation with five applications of electronic von Frey filaments (North Coast Medical, Gilroy, CA, USA). Thermal pain was measured with five applications using Hargreaves' test IITC analgesiometer (IITC Life Sciences, Woodland Hills, CA, USA). Both hot-induced pain and cold-induced pain were measured using a hot/cold plate (IITC Life Sciences, Woodland Hills, CA, USA). Total of eight mice were used in each animal's behavior per group.

### 2.4. Immunohistochemistry

Total of 6 mice were anesthetized with an overdose of choral hydrate and intracardially perfused with saline followed by 4% paraformaldehyde. L3–L5 DRGs were immediately dissected and postfixed with 4% paraformaldehyde. Similar protocols were used as previously described [16]. DRGs were incubated with primary antibodies prepared in blocking solution at 4°C overnight against Nav1.7 (1 : 1000, Alomone), Nav1.8 (1 : 1000, Alomone), and Nav1.9 (1 : 1000, Alomone). The secondary antibodies were goat anti-rabbit (Molecular Probes, Carlsbad, CA, USA). Slides were visualized by use of fluorescence-conjugated secondary antibodies and mounted on cover slips.

### 2.5. Immunoblotting Assay

L3–L5 DRGs from 6 mice were immediately excised to extract proteins. Total proteins were prepared by homogenized DRG as previously described [13]. Peroxidase-conjugated anti-rabbit antibody (1 : 5000) was used as a secondary antibody. The bands were visualized by an enhanced chemiluminescent substrate kit (PIERCE) with LAS-3000 Fujifilm (Fuji Photo Film Co. Ltd). Where applicable, the image intensities of specific bands were quantified with NIH ImageJ software (Bethesda, MD, USA).

### 2.6. Electrophysiology

L3–L5 DRGs were isolated from mice treated with intraplantar saline, CFA, CFA with EA for 4 days. DRG culture and settings for whole-cell patch recording were as previously described [16]. The internal solution contained (in mM) 10 NaCl, 110 CsCl, 20 tetraethylammonium-Cl, 2.5 MgCl_2_, 5 EGTA, 3 Mg^2+^-ATP, and 5 HEPES, adjusted to pH 7.2 with CsOH. The external solution contained (in mM) 100 NaCl, 5 CsCl, 30 tetraethylammonium-Cl, 1.8 CaCl_2_, 1 MgCl_2_, 0.1 CdCl_2_, 25 glucose, 5 4-aminopyridine, and 5 HEPES, adjusted to pH 7.4 with HCl. Osmolarity was adjusted to 300 mosm. Recordings were performed in external solution with 500 nM TTX (Tocris, Avonmouth, UK). TTX-R currents were evoked by a 50 ms test pulse between −70 and 50 mV in 10-mV steps from a holding potential of −70 mV. All recordings were obtained at room temperature (25°C) and completed within 24 h after plating.

### 2.7. Statistical Analysis

All statistic data are presented as the mean ± standard error. Statistical significance between control, inflammation, and EA group was tested using the ANOVA test, followed by a post hoc Tukey's test (*P* < 0.05 was considered statistically significant).

## 3. Results

### 3.1. Inflammatory Pain Models and Behavior

**Figure 1:**
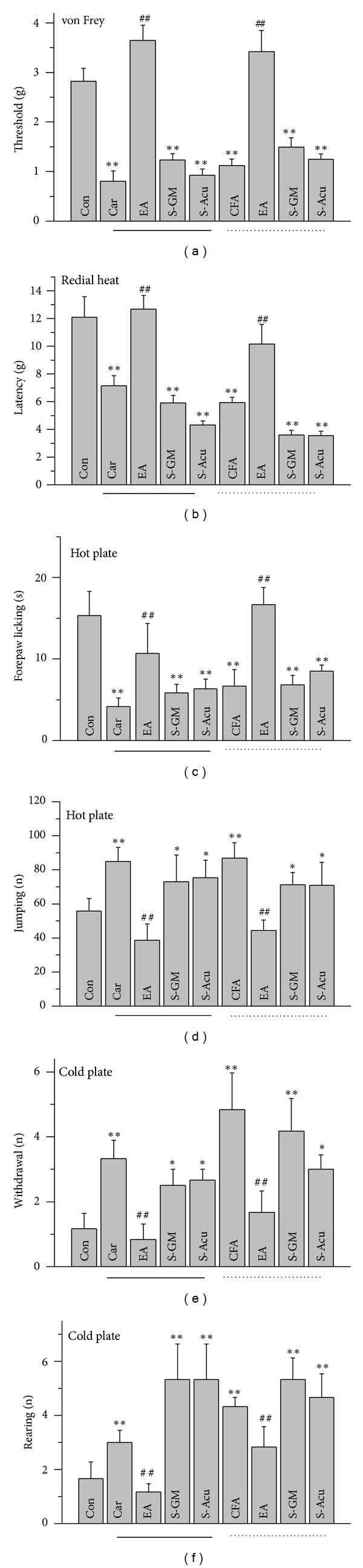
Primary inflammation-induced mechanical and thermal hyperalgesia through carrageenan or CFA injection. (a) Electronic von Frey filament test; (b) radial heat assay; (c) and (d) the latency time for forepaw licking and jumping responses after exposure to a hot plate maintained at 50°C. (c) Latency to first forepaw licking, (d) escape times (jumping) within 5 minutes; (e) and (f) the hind paw withdrawal and rearing on exposure to a cold plate kept at 4°C; (e) withdrawal times; (f) rearing times within 5 minutes. Con: saline, Car: carrageenan and CFA: intraplantar injection into hind paw; Car-EA and CFA-EA: after carrageenan or CFA injection, needles inserted at the ST36 acupoint with electrical stimulation at 2 Hz. *P < 0.05, as compared to that of the baseline. **P < 0.01, as compared to that of the baseline. ^#^ *P* < 0.05; comparison between inflammation and EA-ST36 groups. ^##^ *P* < 0.01; comparison between inflammation and EA-ST36 groups. CFA: complete Freund's adjuvant. Solid lines mean carrageenan-injected group. Dot lines mean CFA-injected group.

We first showed that intraplantar injection of normal saline did not induce mechanical hyperalgesia to be set as a control group (Figure 1(a), 2.82 ± 0.26, *n* = 8). Intraplantar injection of carrageenan or CFA successfully produced mechanical hyperalgesia (Figure 1(a), 0.81 ± 0.21 and 1.12 ± 0.13 of carrageenan and CFA, *n* = 8, *P* < 0.01). Low-frequency 2-Hz EA at ST36 reliably attenuated carrageenan- and CFA-induced hyperalgesia (Figure 1(a), 3.64 ± 0.31 and 3.42 ± 0.43 of carrageenan and CFA, *n* = 8, *P* < 0.01). The phenomenon was not observed neither in S-GM (Figure 1(a), 1.24 ± 0.12 and 1.50 ± 0.18 of carrageenan and CFA, *n* = 8, *P* > 0.05) nor in S-Acu group (Figure 1(a), 0.92 ± 0.13 and 1.25 ± 0.10 of carrageenan and CFA, *n* = 8, *P* > 0.05). We further showed that thermal hyperalgesia was observed in carrageenan-induced inflammatory mice (Figure 1(b), 7.14 ± 0.72 s and 12.1 ± 1.49 s of carrageenan and control, *n* = 8, *P* < 0.01). The same phenomenon was also evoked in CFA-induced inflammatory mice (Figure 1(b), 5.94 ± 0.38, *n* = 8, *P* < 0.01). Both mechanical and thermal hyperalgesia can be reduced by EA at ST36 (Figure 1(b), 12.69 ± 0.97 and 10.17 ± 1.42 of carrageenan and CFA, *n* = 8, *P* < 0.01). The therapeutic effect was not obtained neither in S-GM (Figure 1(b), 5.91 ± 0.54 and 3.59 ± 0.36 of carrageenan and CFA, *n* = 8, *P* > 0.05) nor in S-Acu group (Figure 1(b), 4.31 ± 0.31 and 3.56 ± 0.31 of carrageenan and CFA, *n* = 8, *P* > 0.05).

### 3.2. Thermal Hyperalgesia on the Hot and Cold Plate

Our results displayed that noxious heat can induce thermal pain with a decreased duration of forepaw licking (Figure 1(c), 4.17 ± 1.05 and 6.67 ± 2.01 of carrageenan and CFA, *n* = 8, *P* < 0.01). The phenotype can be attenuated by EA at ST36 (Figure 1(c), 10.67 ± 3.69 and 16.67 ± 2.11 of carrageenan and CFA, *n* = 8, *P* < 0.01). Similar results can also be obtained from criteria regarding jumping analysis. The phenotype was not observed neither in S-GM (Figure 1(c), 5.83 ± 1.07 and 6.83 ± 1.17 of carrageenan and CFA, *n* = 8, *P* > 0.05) nor in S-Acu group (Figure 1(c), 5.60 ± 1.17 and 8.40 ± 1.03 of carrageenan and CFA, *n* = 8, *P* > 0.05). The number of jumping instances increased after inflammation treatment (Figure 1(d), 84.83 ± 8.37 and 86.83 ± 9.09 of carrageenan and CFA, *n* = 8, *P* < 0.01). Both carrageenan- and CFA-induced thermal pain can be further ameliorated by EA stimulation (Figure 1(d), 38.67 ± 9.49 and 44.5 ± 6.09 of carrageenan and CFA, *n* = 8, *P* < 0.01). The effect was not obtained neither in S-GM (Figure 1(d), 66.50 ± 5.6 and 69.60 ± 18.74 of carrageenan and CFA, *n* = 8, *P* > 0.05) nor in S-Acu group (Figure 1(d), 68.75 ± 5.43 and 68.40 ± 12.91 of carrageenan and CFA, *n* = 8, *P* > 0.05). Our results also show that cold hyperalgesia was induced by carrageenan, and CFA intraplantar injection was analyzed with hind paw withdrawal number (Figure 1(e), 3.33 ± 0.56 and 4.83 ± 1.14 of carrageenan and CFA, *n* = 8, *P* < 0.01). Accordingly, similar curative effects of EA were observed in both carrageenan- and CFA-induced inflammatory mice (Figure 1(e), 0.83 ± 0.48 and 1.67 ± 0.67 of carrageenan and CFA, *n* = 8, *P* < 0.01). The effect was not obtained neither in S-GM (Figure 1(e), 2.50 ± 0.50 and 4.20 ± 1.24 of carrageenan and CFA, *n* = 8, *P* > 0.05) nor in S-Acu group (Figure 1(e), 2.67 ± 0.33 and 3.0 ± 0.45 of carrageenan and CFA, *n* = 8, *P* > 0.05). Cold hyperalgesia was induced by carrageenan, and CFA intraplantar injection was analyzed with rearing number (Figure 1(f), 3.0 ± 0.45 and 4.33 ± 0.33 of carrageenan and CFA, *n* = 8, *P* < 0.01). Accordingly, similar curative effects of EA were observed in both carrageenan- and CFA-induced inflammatory mice (Figure 1(e), 1.17 ± 0.31 and 2.83 ± 0.75 of carrageenan and CFA, *n* = 8, *P* < 0.01). The effect was not obtained neither in S-GM (Figure 1(f), 4.60 ± 1.29 and 5.0 ± 0.80 of carrageenan and CFA, *n* = 8, *P* > 0.05) nor in S-Acu group (Figure 1(f), 5.33 ± 1.31 and 4.67 ± 0.88 of carrageenan and CFA, *n* = 8, *P* > 0.05).

### 3.3. Immunohistochemistry Expression of Navs in DRG Neurons

**Figure 2:**
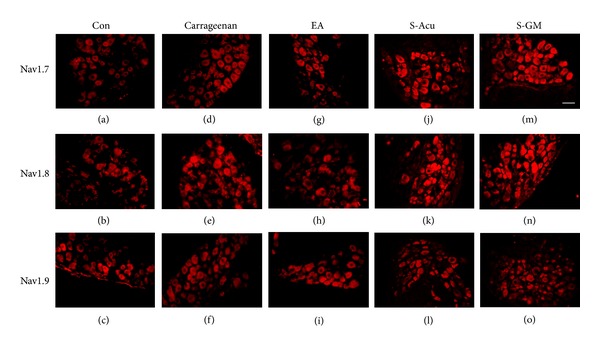
Nav1.7 and Nav1.8 expressions were increased in ipsilateral DRGs after intraplantar carrageenan injection and further attenuated by EA at the ST36 acupoint in mice, though Nav1.9 was not different. (a)–(c) Nav1.7, Nav1.8, and Nav1.9 immunoreactive neurons were found in lumbar DRGs at the ipsilateral site of the saline-injected group. (d)-(e) Nav1.7 and Nav1.8 immunoreactive neurons were increased in the carrageenan-injected group, but (f) Nav1.9 immunoreactive neurons were not increased. (g)-(h) Carrageenan-induced increases of Nav1.7 and Nav1.8 were attenuated by EA, as compared to those of the carrageenan-induced group. (i) Nav1.9 immunoreactive neurons were not altered by EA at the ipsilateral site of inflammation. (j)-(k) Nav1.7 and Nav1.8 immunoreactive neurons were increased in the S-Acu group. (l) Nav1.9 immunoreactive neurons were not altered in the S-Acu group. (m)-(n) Nav1.7 and Nav1.8 immunoreactive neurons were increased in the S-GM group. (o) Nav1.9 immunoreactive neurons were not altered in the S-GM group. Scale bar = 50 um.

**Figure 3:**
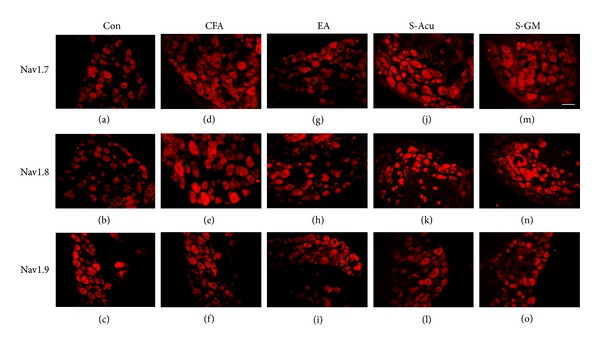
Nav1.7 and Nav1.8 expressions were increased in ipsilateral DRGs after intraplantar CFA injection and further attenuated by EA at the ST36 acupoint in mice, though Nav1.9 was not different. (a)–(c) Nav1.7, Nav1.8, and Nav1.9 immunoreactive neurons were found in lumbar DRGs at the ipsilateral site of the saline-injected group. (d)-(e) Nav1.7 and Nav1.8 immunoreactive neurons were increased in the CFA-injected group, but (f) Nav1.9 immunoreactive neurons were not increased. (g)-(h) CFA-induced increases of Nav1.7 and Nav1.8 were attenuated by EA, as compared to those of the CFA-induced group. (i) Nav1.9 immunoreactive neurons were not altered by EA at the ipsilateral site of inflammation. (j)-(k) Nav1.7 and Nav1.8 immunoreactive neurons were increased in the S-Acu group. (l) Nav1.9 immunoreactive neurons were not altered in the S-Acu group. (m)-(n) Nav1.7 and Nav1.8 immunoreactive neurons were increased in the S-GM group. (o) Nav1.9 immunoreactive neurons were not altered in the S-GM group. Scale bar = 50 um.

Our data showed that Nav1.7 sodium channels were distributed in L3–L5 DRG neurons (Figures 2(a) and 3(a)). Intraplantar injection of carrageenan or CFA reliably increased the expression of Nav1.7 sodium channels in L3–L5 DRG neurons (Figures 2(d) and 3(d)). Dramatically, Nav1.7 channels were negatively regulated to a normal level by applying 2 Hz EA treatment at ST36 acupoint (Figures 2(g) and 3(g)). EA-elicited downregulation of Nav 1.7 was not observed neither in sham-Acu (Figures 2(j) and 3(j)) nor in sham-GM groups (Figures 2(m) and 3(m)). Our results also show that Nav1.8 channels were expressed in DRG neurons in saline-injected neurons (Figures 2(b) and 3(b)). With the injection of carrageenan or CFA, Nav1.8 channels were greatly increased in DRG neurons (Figures 2(e) and 3(e)). The phenomenon was similar to previous results [2]. Importantly, 2 Hz EA at ST36 significantly reverses the overexpression of Nav1.8 channels in DRG neurons (Figures 2(h) and 3(h)). The effects were not obtained from sham-Acu (Figures 2(k) and 3(k)) and sham-GM groups (Figures 2(n) and 3(n)). The expression on Nav1.9 channels was observed in control group (Figures 2(c) and 3(c)). In carrageenan- and CFA-induced inflammation group, the expression of Nav1.9 was similar to that of the control one suggesting absence of the role of Nav1.9 in this model (Figures 2(f) and 3(f)). Similarly, the expression of Nav1.9 channels was not significantly different in the EA-treated group (Figures 2(i) and 3(i)), sham-Acu (Figures 2(l) and 3(l)), and sham-GM groups (Figures 2(o) and 3(o)).

### 3.4. Immunoblotting Quality of Navs in DRG Neurons

**Figure 4:**
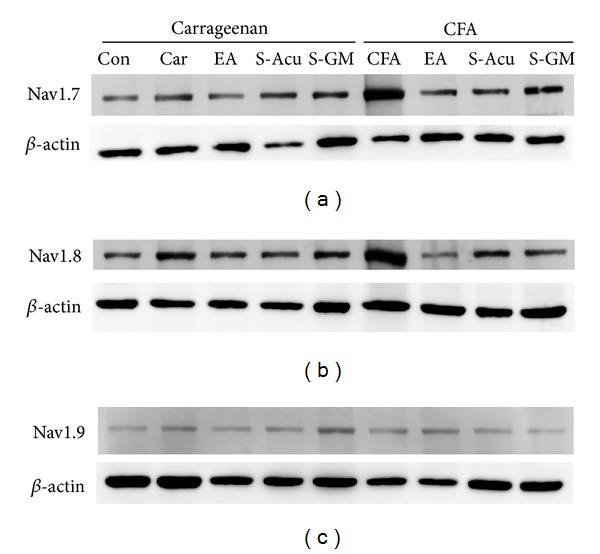
Nav1.7 and Nav1.8 protein levels were increased in lumbar DRGs in both intraplantar carrageenan- and CFA-induced inflammation and further attenuated by EA at the ST36 acupoint in mice, but Nav1.9 proteins were not altered. (a) DRGs lysates were immunoreactive with specific antibodies to Nav1.7 and a substantially increased signal at the ipsilateral site, as compared to that of the saline-injected group. Nav1.7 protein levels were attenuated by EA at the ST36 acupoint, as compared to that of the carrageenan- and CFA-induced groups. (b) Nav1.8 displayed similar results to Nav1.7. The protein levels of S-Acu and S-GM were similar to inflamed but not EA group. (c) Nav1.9 protein levels were not changed in both the carrageenan- and CFA-injected sites. Nav1.9 protein levels were not attenuated by EA at the ST36 acupoint, as compared to those of the carrageenan- and CFA-induced groups, either. Nav1.9 proteins were not altered at the ipsilateral site of inflammation and EA stimulation.

**Figure 5:**
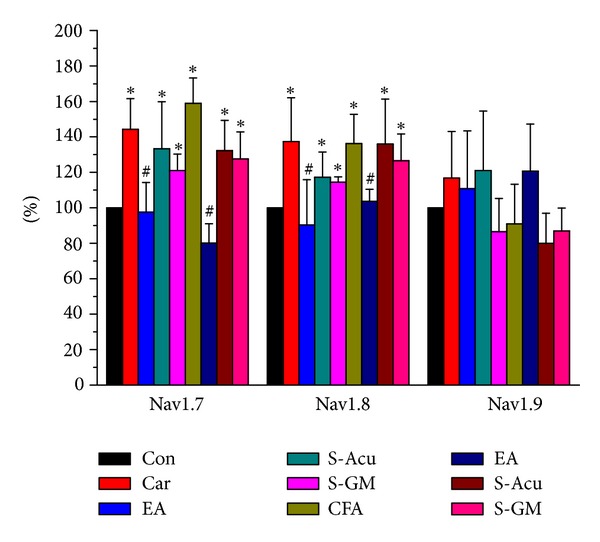
Protein levels of Nav1.7, Nav1.8, and Nav1.9 in the L3–L5 DRGs in mice in control, Car, EA, S-Acu, S-GM, CFA, EA, S-Acu, S-GM groups. The percentage of Nav protein levels from lumbar DRGs was presented in each group. *P < 0.05, as compared to control group. ^#^ *P* < 0.05; comparison between inflammation and EA groups.

We further showed that Nav1.7 and Nav1.8 channels were increased during carrageenan- and CFA-induced inflammatory pain in mice L3–L5 DRGs by using western blot technique (Figures 4(a) and 4(b)). In contrast, the expression of Nav1.9 sodium channels was not changed in this condition (Figure 4(c)). Our results suggested that Nav1.7 channels were attenuated by 2 Hz EA at ST36 acupoint in carrageenan- and CFA-induced inflammation pain (Figure 4(a) displayed a 46.2% decrease in the signal, as compared with the carrageenan group, *n* = 6, *P* < 0.05; a 78.8% decrease in the signal, as compared with the CFA group, *n* = 6, *P* < 0.05). Similar results were observed in Nav1.8 (Figure 4(b) displayed a 24.8% decrease in the signal, compared with the carrageenan group, *n* = 6, *P* < 0.05; a 30.7% decrease in the signal, compared with the CFA group, *n* = 6, *P* < 0.05). The protein levels of S-Acu and S-GM were similar to inflamed but not EA group suggesting acupoint specificity. Nav1.9 displayed no significant difference per group (Figure 4(c)). Accordingly, our results suggest that 2-Hz EA at the ST36 acupoint has the ability to ameliorate carrageenan- and CFA-induced overexpression of Nav1.7 and Nav1.8, rather than Nav1.9 sodium channels. All data were analyzed and presented in Figure 5.

### 3.5. Functional Analysis of TTX-R Currents Using Whole-Cell Recording

**Figure 6:**
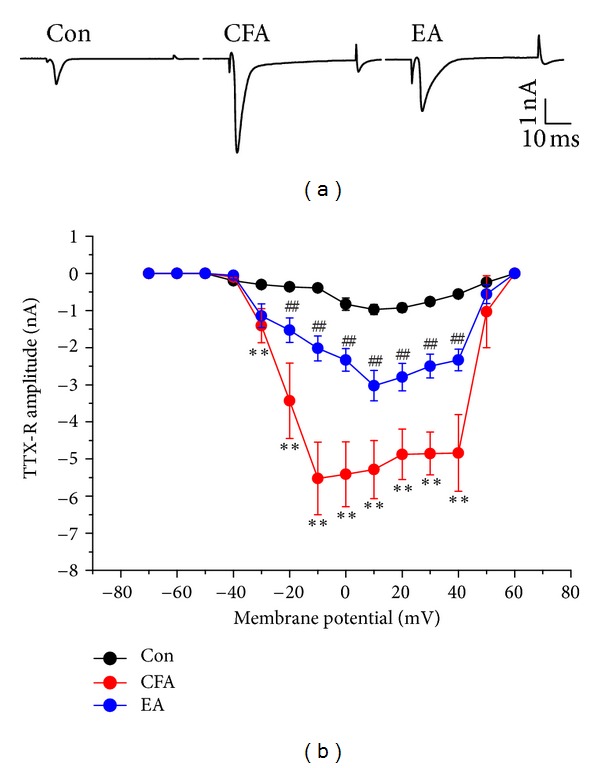
Tetrodotoxin-resistant (TTX-R) sodium currents in L3–L5 DRG neurons. (a) Representative TTX-R current traces in Con, CFA, and EA groups. The TTX-R currents were induced by membrane potential depolarized to −40 mV. (b) Mean peak amplitudes of TTX-R currents in each group. **P < 0.01 compared to control group. ^##^ *P* < 0.01 compared to CFA group.

To determine whether EA attenuates the neuronal excitability after CFA-induced inflammation pain model, we used whole-cell recording to record the TTX-R sodium currents in small-to-medium-size (<34 *μ*m) DRG neurons. In control group, TTX-R currents were obtained with membrane potential depolarized to −40 mV. However, intraplantar inflammation by CFA injection potentiated the amplitudes of TTX-R currents in DRG neurons. The potentiation of TTX-R currents was decreased in DRG neurons obtained from EA-treated group (Figure 6(a)). The relationship between membrane potential and Nav currents was plotted in Figure 6(b).

## 4. Discussion

In this study, we first established animal models of inflammatory pain by injection of carrageenan or CFA into hind paw. Animals with inflammatory pain showed mechanical and thermal hyperalgesia using a von Frey filament test, Hargreaves' test, and hot/cold plate tests. EA stimulation at the ST36 acupoint reduced inflammatory hyperalgesia in both carrageenan and CFA groups. Our results indicated that Nav1.7 and 1.8, but not Nav1.9, were upregulated in both carrageenan and CFA-induced hyperalgesia, which suggested the important role of Nav1.7 and 1.8 in inflammatory pain. We showed that EA at Zusanli (ST36) acupoint at 2 Hz low-frequency stimulation reduced pain thresholds accompanied by decreasing the expression of Nav1.7 and 1.8, rather than Nav1.9, sodium channels in DRG neurons.

Zhang et al. reported that EA at 10 Hz frequency significantly reduced CFA-induced hind paw edema. Moreover, EA attenuates inflammatory response through the hypothalamus-pituitary-adrenal axis (HPA) and the nervous system [14]. Recently, EA also suppresses the expression of neurokinin-1 in spinal cord dorsal horn induced by inflammation in rats [15]. These phenomena were not observed in sham control groups suggesting the acupoint-specific effect [14, 15]. Our results were consistent with these studies that the antinociceptive effect was only observed in EA but not in sham-Acu and sham-GM groups.

Numerous studies have investigated the role of different Navs in pain, neuron excitability, and action potential firing [4, 17]. The most emphatic evidence implicating a specific ion channel participating in pain comes from studies on complete insensitivity to pain using gene knock mice [18, 19]. Nav1.7 was greatly expressed in C-fiber free nerve endings, playing a crucial role in nociceptive information [20]. Recent studies have strongly supported Navs as potential analgesic drugs, according to antisense and knockout mice [6, 21]. Derivatives from benzazepine and imidazopyridine were also developed to block Nav1.7 channels for pain treatment [22]. Our results clearly indicate that EA reliably attenuated carrageenan- and CFA-induced inflammation pain by ameliorating Nav1.7 overexpression. This is the first paper regarding the functional role of acupuncture in pain manipulation and its novel findings pertaining to Nav1.7 channel alteration.

Chronic intrathecal Nav1.8 antisense injection successfully attenuated the Nav1.8-induced current and decreased mechanical allodynia after intraplantar CFA injection [23]. Developing a specific Navs channel blocker is possible for inflammatory pain. A-803467 is a novel specific blocker for the Nav1.8 channel and can ameliorate inflammatory pain in rats [24]. Nav1.9 is a TTX-R sodium channel greatly expressed in small diameter C-fibers and contributes to membrane properties, particularly in nociceptive neurons [25]. Nav1.9 is also suggested to regulate inflammatory pain thresholds [26]. Animal behavior studies have also demonstrated that deletion of Nav1.9 channel expression prevents inflammatory mediator-induced hyperalgesia [27, 28]. Inflammatory mediators, such as PGE_2_, can reliably increase the Nav1.9 channel current in mice DRG neurons with G-protein activation [29]. Our data provide highly valuable results from investigating inflammation pain regarding ancient acupuncture mechanisms that can be further applied to clinical medicine.
